# Quantitative stability of MGMT promoter methylation in recurrent glioma and implications for temozolomide rechallenge

**DOI:** 10.1007/s11060-026-05778-y

**Published:** 2026-09-16

**Authors:** Henry Noren, Gabriella Pelofsky, Brianna Suffren, Laura Mittelman, Christine Yohn, Aminah Twyman, Christopher A. Febres-Aldana, Arevik Abramyan, Aparna Sertil, Randy S. D’Amico, Jonathan H. Sherman, Morana Vojnic

**Affiliations:** 1 Department of Neurosurgery, Rutgers Robert Wood Johnson Medical School, 195 Little Albany Street, New Brunswick, NJ 08901 USA; 2 Department of Neurosurgery, Rutgers New Jersey Medical School, Newark, NJ USA; 3 Department of Neurosurgery, Lenox Hill Hospital, New York, NY USA; 4 Laboratory of Pathology, National Cancer Institute, National Institutes of Health, Bethesda, MD USA; 5 Caris Life Sciences, Phoenix, AZ USA; 6 Rutgers Cancer Institute, New Brunswick, NJ USA; 7 RWJBarnabas Health, West Orange, NJ USA

**Keywords:** Recurrent Glioma, MGMT promoter methylation, Temozolomide, Epigenetics, Molecular dynamics

## Abstract

**Purpose:**

O^6^-methylguanine–DNA methyltransferase (MGMT) promoter methylation is a key predictive biomarker for temozolomide (TMZ) response in glioma. MGMT status is routinely assessed at diagnosis; however, its utility at recurrence remains incompletely characterized. We aimed to quantify MGMT methylation stability and assess the clinical relevance of reported status changes in recurrent glioma.

**Methods:**

We analyzed paired tumor samples from 426 patients with recurrent glioma in a large commercial molecular database containing quantitative methylation percentage and binary methylation status at two sample time points. A separate multi-institutional clinical cohort (*n* = 27) with detailed treatment annotation was analyzed to explore associations between MGMT status at recurrence and treatment decision-making. Statistical analyses assessed quantitative methylation dynamics and time between samples as a surrogate for disease course.

**Results:**

MGMT status remained unchanged in 84% of tumors across recurrence. 16% demonstrated a status change, commonly among tumors with low baseline methylation near classification thresholds. Longitudinal quantitative methylation changes were modest overall. Tumors that remained, gained, or lost hypermethylation demonstrated longer intervals between samples compared to consistently unmethylated tumors (*p* < 0.01). In the clinical cohort, TMZ rechallenge was more frequently pursued in patients retaining hypermethylation at recurrence.

**Conclusion:**

MGMT promoter methylation is quantitatively stable in the majority of recurrent gliomas. Status changes predominantly occur in tumors with borderline methylation, highlighting the limitations of binary classification. These findings support routine reassessment of MGMT at recurrence and underscore the value of quantitative reporting to better inform therapeutic decision-making, particularly regarding TMZ rechallenge.

## Introduction

Diffuse gliomas comprise a heterogeneous group of primary central nervous system tumors characterized by wide variability in biological behavior, treatment response, and clinical outcome. Despite advances in molecular classification, which have improved prognostic stratification and therapeutic decision-making, outcomes for high-grade glioma remain poor despite aggressive multimodal therapy [1, 2]. Standard treatment typically consists of maximal safe surgical resection followed by radiotherapy with concurrent and adjuvant temozolomide (TMZ) [1]. Methylation of the O^6^-methylguanine–DNA methyltransferase (MGMT) promoter is an important predictor of response to alkylating chemotherapy [2–4]. Specifically, MGMT promoter methylation is associated with improved progression-free and overall survival in patients treated with TMZ [5–9].

While MGMT methylation status is routinely assessed at diagnosis, its stability over the course of disease and particularly at recurrence has important implications for treatment selection but remains incompletely defined. Prior studies examining paired primary and recurrent glioma samples have generally demonstrated high concordance of MGMT methylation status, with reported stability rates ranging from approximately 70–85% [10–13]. A clinically meaningful minority of tumors exhibit apparent gains or losses of MGMT hypermethylation at recurrence, raising questions about epigenetic evolution under therapeutic pressure and the reliability of initial MGMT assessment for guiding re-treatment decisions [10–14]. However, these studies have largely reported binary methylation classification, making it difficult to determine if changes reflect biological effects or variation close to established categorical thresholds. In addition, these studies have been limited by relatively small sample sizes and have not investigated how quantitative methylation dynamics may influence clinical decision-making.

MGMT methylation is increasingly recognized as a quantitative continuum rather than a strict dichotomy [15–18]. Tumors with methylation levels near established cutoffs may be particularly vulnerable to classification variability driven by intratumoral heterogeneity, sampling differences, assay methodology, or modest epigenetic drift over time [15–18]. Such borderline cases may account for many reported status transitions and demonstrate less predictable treatment response, underscoring the limitations of binary reporting. Beyond molecular considerations, MGMT status may influence therapeutic decision-making at recurrence, particularly regarding rechallenge with TMZ. Clinical trials and retrospective analyses suggest that patients with retained MGMT hypermethylation derive greater benefit from TMZ rechallenge, whereas those with unmethylated tumors are more often directed toward alternative systemic therapies, clinical trials, re-irradiation, or supportive care [19, 20]. However, real-world data examining how MGMT methylation dynamics are incorporated into treatment decisions at recurrence, especially outside of clinical trial populations, remain limited.

In this study, we aimed to address these gaps by evaluating both the reported categorical and quantitative stability and longitudinal dynamics of MGMT methylation using one of the largest paired cohorts of recurrent glioma reported to date with such data. Because clinical decision-making continues to rely largely on binary status classification, we first characterized longitudinal categorical MGMT transition patterns. Quantitative methylation percentages were then analyzed to determine whether apparent binary status transitions reflected epigenetic shifts or variability near established classification thresholds. In a complementary multi-institutional clinical cohort with detailed treatment annotation, we further explored how MGMT status at recurrence relates to real-world treatment decision-making, particularly the use of TMZ rechallenge. Together, these analyses seek to clarify the clinical relevance of MGMT methylation reassessment at recurrence and to inform more nuanced, quantitatively informed precision treatment strategies in recurrent glioma.

## Methods

### Commercial molecular database cohort

#### Patient selection

Data was obtained from a proprietary molecular profiling database maintained by Caris Life Sciences comprising tumor specimens submitted for routine clinical molecular testing from multiple institutions. The database includes molecular profiling results linked to basic specimen information but does not contain detailed longitudinal clinical or treatment information. Patients with recurrent glioma who underwent molecular testing at two distinct time points were identified, initially generating a cohort of 533 patients. Patients were excluded if MGMT promoter methylation data were unavailable for either time point due to insufficient tissue or DNA quantity. After exclusion, 426 patients with complete paired MGMT methylation data were included in the final analytic cohort.

#### MGMT promoter methylation assessment

MGMT promoter methylation was assessed by pyrosequencing of CpG sites 74–78 using DNA extracted from formalin-fixed paraffin-embedded tumor tissue (≥ 50% tumor nuclei). Following bisulfite conversion, target regions in exon 1 were amplified and analyzed on a pyrosequencing platform. Methylation was reported by the testing laboratory as both a quantitative methylation percentage and a categorical status of hypermethylated (≥ 9%), equivocal (7-<9%), or unmethylated (< 7%). Samples reported as equivocal methylation were assigned as unmethylated for analysis, in accordance with the approach used in previous studies [15–18]. Quantitative methylation percentages were retained for all analyses to evaluate methylation dynamics independent of binary classification.

#### Definition of methylation transition groups

Patients were categorized into four longitudinal MGMT methylation groups based on categorical status at the first and second sample time points: (1) remained unmethylated; (2) transitioned from unmethylated to hypermethylated; (3) remained hypermethylated; and (4) transitioned from hypermethylated to unmethylated. These categorical transitions were assessed because treatment decisions are often based on categorical MGMT status, whereas quantitative methylation percentages were analyzed to provide biological context for observed transitions.

#### Tumor location and time between surgeries

Primary tumor location of the first sample was reported as frontal, temporal, parietal, occipital, overlapping regions, or unspecified/other. The interval between tissue collection time points was calculated as the number of months between the paired samples. This interval was used as a surrogate measure of disease course, acknowledging that it reflects both biological behavior and treatment response rather than a direct measure of progression-free survival. To reduce misclassification related to early postoperative interventions or sampling artifacts, analyses involving the interval between samples were restricted to samples collected at least three months apart.

### Clinical cohort

#### Patient identification and data collection

A retrospective clinical cohort of 27 adult patients with recurrent glioma was identified from two academic institutions (Rutgers Cancer Institute/RWJBarnabas Health and Lenox Hill Hospital). Ten patients overlapped with the commercial molecular database cohort. Eligible patients were ≥ 18 years old and had undergone at least two surgical procedures for glioma separated by a minimum of six months, with MGMT promoter methylation assessed on both tumor specimens.

Clinical variables collected included age at diagnosis, tumor type and grade, MGMT promoter methylation status at each time point, treatment modalities (temozolomide, radiation therapy, investigational therapy, or supportive care), and time between surgeries. MGMT promoter methylation status was extracted from clinically reported pathology or molecular profiling reports available on the electronic medical record. Testing was performed as part of routine clinical care by the reporting laboratory and no additional testing was performed for this study. Methylation reported as “low” in one subject was treated as unmethylated, consistent with the handling of equivocal cases in the commercial cohort. The available records did not consistently report if recurrence was detected during active adjuvant TMZ therapy or the treatment-free interval prior to TMZ rechallenge.

#### Purpose of the clinical cohort

The purpose of the commercial molecular database was to characterize longitudinal quantitative MGMT methylation dynamics in a large cohort, whereas the institutional clinical cohort provided detailed treatment data and clinical context unavailable in the commercial database. The clinical cohort was analyzed in an exploratory, hypothesis-generating manner to assess real-world treatment decision-making at recurrence, particularly TMZ rechallenge relative to MGMT methylation status. This cohort was not powered for survival analyses or causal inference regarding treatment efficacy.

### Statistical analysis

Descriptive statistics were used to summarize patient characteristics, methylation status distributions, and quantitative methylation percentages. Mean values were reported for continuous variables. In the commercial cohort, two-sample t-tests were used to compare quantitative methylation percentages between groups when distributions approximated normality; nonparametric tests were used when appropriate. Analysis of variance (ANOVA) was used to compare initial methylation percentages and time between surgeries across the four methylation transition groups. Two-proportion z-tests were used to assess differences in the distribution of methylation transition groups across tumor locations. Statistical significance was defined as a two-sided p-value < 0.05. Analyses were performed using SciPy (version 1.17.1) and statsmodels (version 0.14.6) in Python (version 3.12).

### Ethical considerations

All data were de-identified prior to analysis. Institutional review board approval was obtained and the study was conducted in accordance with institutional and ethical guidelines for retrospective research.

## Results

### Commercial molecular database cohort

#### Patient characteristics and MGMT methylation in first sample

Among 426 patients with paired MGMT promoter methylation data at two time points, methylation status was reported in the database as unmethylated, equivocal, or hypermethylated. Among tumors designated as hypermethylated at the first time point, the mean quantitative methylation percentage was 35.6% (range 9–89%). Tumors designated as unmethylated demonstrated low methylation levels (mean 2.1%, range 0–6%), while equivocal cases clustered near classification thresholds (mean 7.3%, range 7–8%). For binary analysis, equivocal cases were treated as unmethylated, consistent with the analytic approach defined in the Methods.

#### Longitudinal stability of MGMT promoter methylation

MGMT promoter methylation status remained unchanged between initial collection and recurrent tumor samples in the majority of cases. Overall, 84% (358/426) of tumors retained the same categorical MGMT status at recurrence, with 52% (223/426) remaining unmethylated and 32% (135/426) remaining hypermethylated. 16% (68/426) of tumors demonstrated a categorical status change, including 11% (46/426) transitioning from hypermethylated to unmethylated and 5% (22/426) transitioning from unmethylated to hypermethylated.

Despite these categorical transitions, quantitative methylation changes across the cohort were modest. The mean change in methylation percentage between the first and second samples was − 1.4 percentage points (SD 16.0). Tumors that remained unmethylated or hypermethylated demonstrated minimal average change in methylation percentage (–0.3 and + 0.6 points, respectively), whereas tumors that changed categorical status exhibited larger absolute shifts.

#### Quantitative methylation levels and apparent status transitions

Apparent MGMT status transitions were strongly associated with borderline quantitative methylation levels, defined here as values near the categorical thresholds where modest differences in methylation percentage may result in a different classification. Among tumors that were hypermethylated initially, those that transitioned to unmethylated had baseline methylation percentages that were on average 13.5 percentage points lower than tumors that remained hypermethylated (*p* < 0.001). Similarly, those that transitioned from unmethylated to hypermethylated had recurrence methylation percentages that were on average 16.3 percentage points lower than tumors that remained consistently hypermethylated (*p* < 0.001). These findings indicate that most categorical status changes occurred near classification thresholds and were driven by quantitative variability rather than true epigenetic reprogramming.

#### Tumor location and MGMT methylation dynamics

**Fig. 1:**
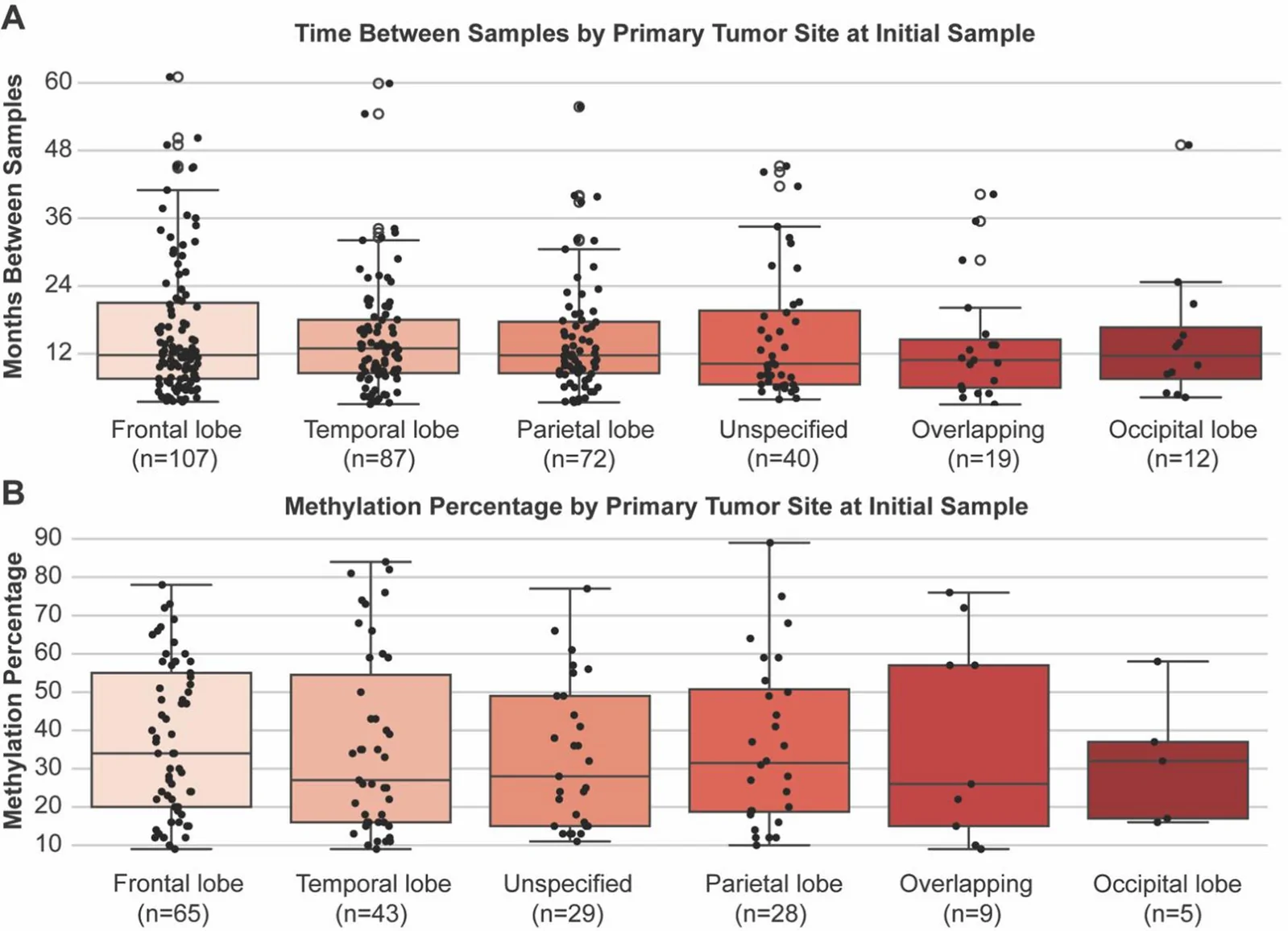
Regional effects on methylation and interval between samples. (**A**) Time interval between first and second sample by tumor site. The interval between samples, used as a surrogate for time to recurrence, was comparable among the six main primary tumor sites reported with a mean of about 12 months after initial collection. (**B**) Quantitative methylation percentage by tumor sites, excluding unmethylated tumors. There were no significant differences in methylation % among those designated as hypermethylated, with a mean of 35%

Primary tumor location at initial collection was frontal in 31% of cases, temporal in 25%, parietal in 19%, occipital in 4%, overlapping regions in 5%, and unspecified or other in 16%. 62% of tumors recurred in the same anatomical region as the initial collection. There were no significant differences in the interval between samples by location among patients with samples collected at least three months apart (*p* = 0.28; Fig. 1A), nor were there significant differences in quantitative MGMT methylation percentage at initial collection across tumor locations (*p* = 0.39; Fig. 1B).

**Fig. 2:**
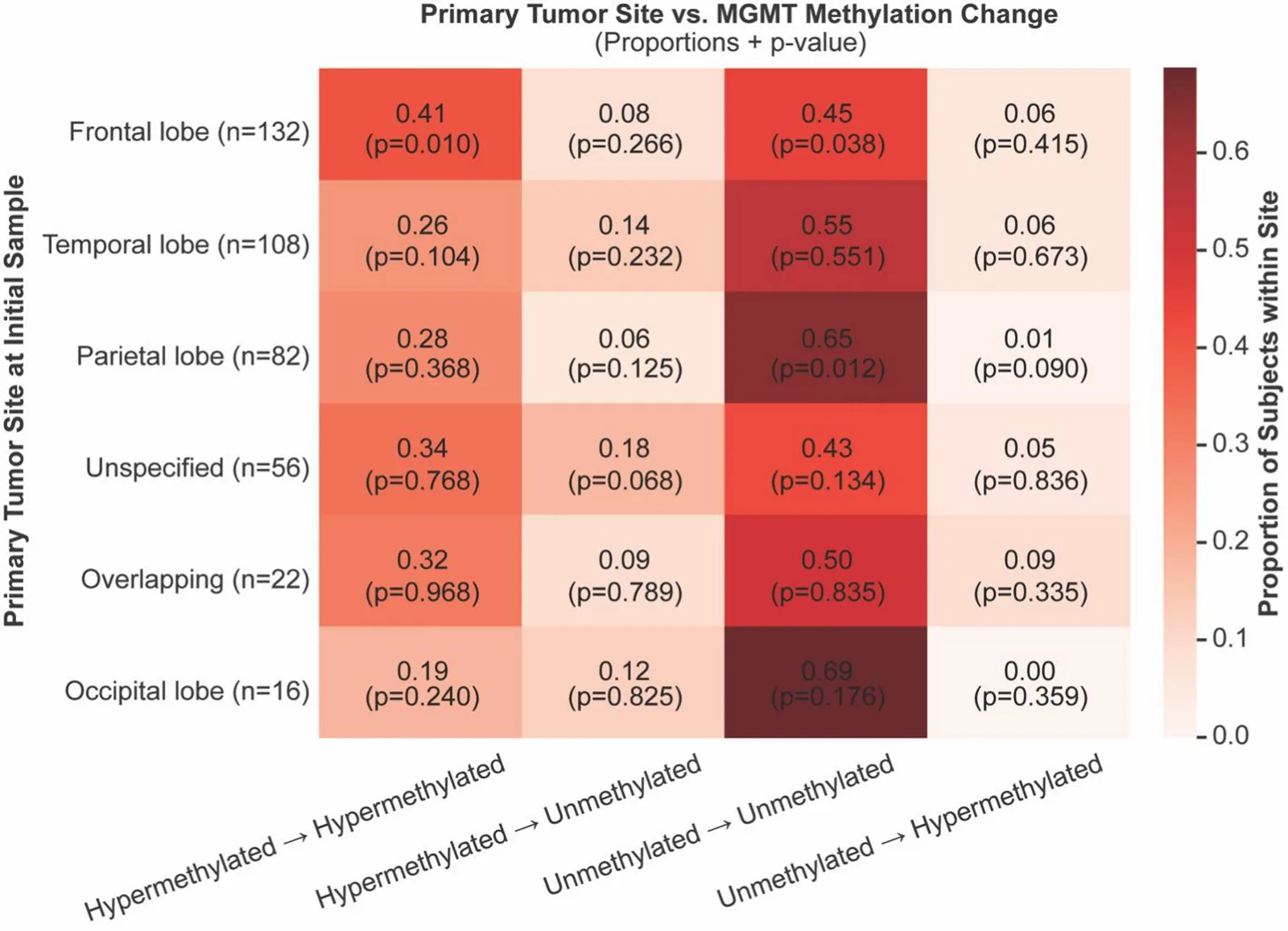
Proportion of methylation changes by tumor sites. The proportion of patients in each of the four methylation categories was comparable among most primary tumor sites. Compared with all other tumor sites combined, gliomas of the frontal lobe had a significantly higher proportion of tumors remaining hypermethylated (*p* = 0.01) and a lower proportion of tumors remaining unmethylated (*p* = 0.038, proportion z-test)

However, tumors originating in the frontal lobe demonstrated a distinct distribution of methylation stability. For each tumor site, the proportion of tumors in each methylation category was compared with that of all other tumor sites combined. Frontal lobe tumors were more likely to remain hypermethylated at recurrence (41%) and less likely to remain unmethylated (45%) compared with tumors in other regions (*p* = 0.01 and *p* = 0.038 respectively, proportion z-test; Fig. 2). The proportions of tumors demonstrating categorical methylation transitions were otherwise similar across anatomical locations.

#### Time interval between samples and MGMT methylation groups

**Fig. 3:**
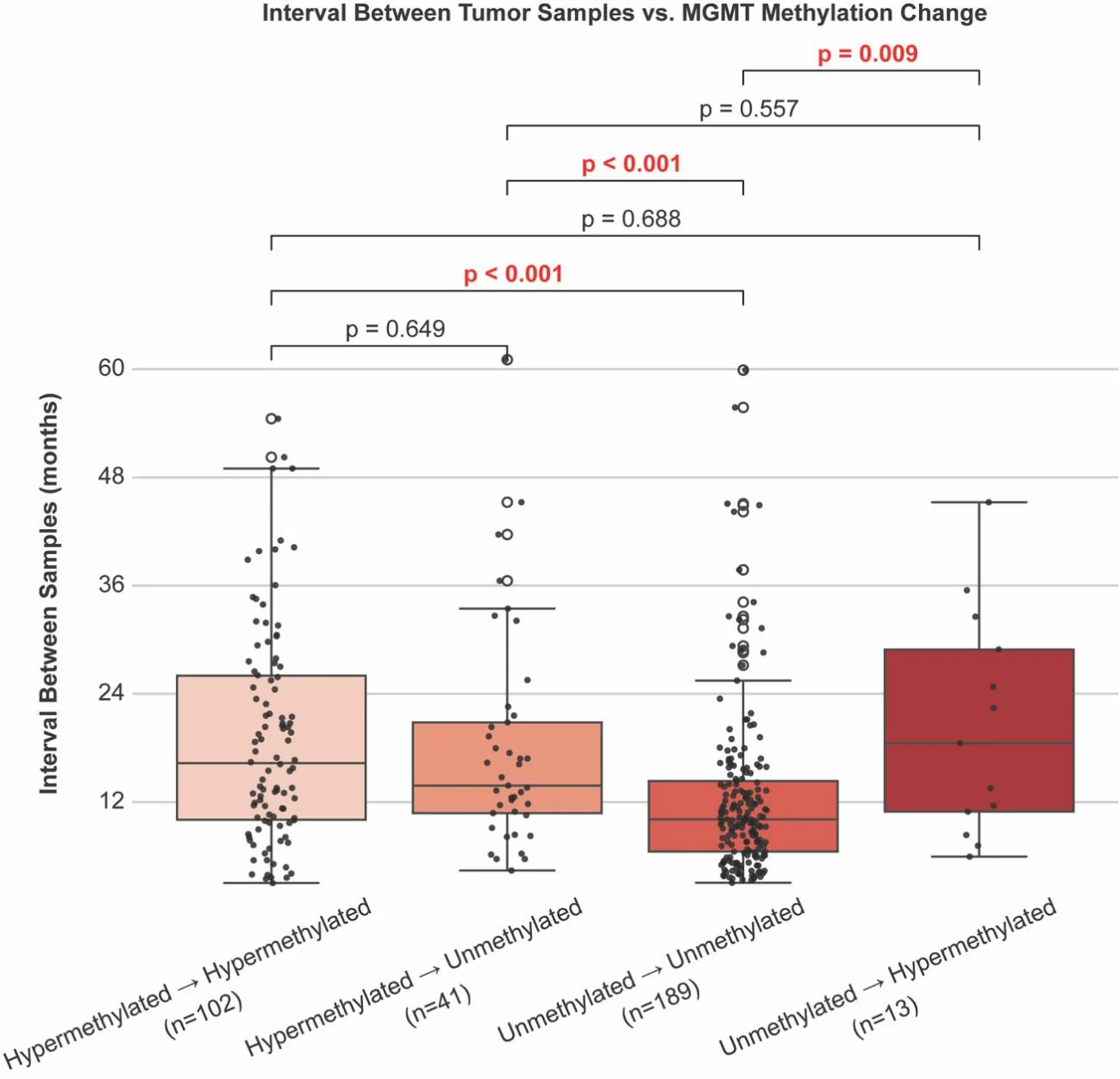
Time interval between samples by methylation transition. Tumors that remained unmethylated demonstrated a significantly shorter interval between samples than those that remained hypermethylated or were originally hypermethylated (*p* < 0.001). Tumors that gained hypermethylation had a significantly longer interval between samples than tumors that remained unmethylated (*p* = 0.009)

Among patients with at least three months between collections, the interval between samples differed by MGMT methylation group (Fig. 3). Tumors that remained hypermethylated, lost hypermethylation, or gained hypermethylation all demonstrated longer intervals between samples compared with tumors that remained unmethylated. Specifically, the interval was 6.7 months longer for consistently hypermethylated tumors and 5.6 months longer for tumors that lost hypermethylation compared with consistently unmethylated tumors (both *p* < 0.001). Tumors that gained hypermethylation also demonstrated a longer interval between samples than consistently unmethylated tumors (difference 8.2 months, *p* = 0.009). These differences reflect expected associations between MGMT methylation status and disease course and were interpreted descriptively as a surrogate for underlying tumor biology and treatment responsiveness.

### Clinical cohort

#### Patient characteristics

The clinical cohort consisted of 27 adult patients with recurrent glioma treated at two academic institutions (Table 1). The mean age at diagnosis was 51.1 years (SD 15.3), with no significant age differences across MGMT methylation transition groups. The majority of tumors were glioblastoma (74.1%), followed by astrocytoma (grades II–III, 22.2%) and one gliosarcoma (3.7%). The mean interval between surgeries was 17.8 months (SD 14.4) with no significant differences across methylation groups.

#### Treatment patterns at recurrence

Nearly all patients (96.3%) received TMZ following initial surgery, regardless of MGMT status. At recurrence, 44.4% of patients were re-treated with TMZ. Rechallenge with temozolomide was more frequently pursued in patients whose tumors remained hypermethylated (54.5%) compared with those that remained unmethylated (41.7%), although these differences were not statistically significant in this small cohort. Radiation therapy was administered to 96.3% of patients after initial surgery, while 29.6% underwent repeat radiation at recurrence. Repeat radiation was more commonly used in patients with consistently unmethylated tumors than in those who remained hypermethylated, demonstrating an inverse pattern relative to temozolomide rechallenge.

These exploratory findings describe differences in treatment selection according to MGMT promoter methylation status at recurrence, particularly with respect to the selection of alkylating chemotherapy versus alternative therapeutic strategies, in which TMZ rechallenge was more commonly pursued in patients whose tumors remained hypermethylated.

**Table 1:** Clinical cohort characteristics

		Overall	Hypermethylated to Hypermethylated	Hypermethylated to Unmethylated	Unmethylated to Unmethylated	Unmethylated to Hypermethylated
*n*		27	11	2	12	2
Age at diagnosis (years), mean (SD)		51.1 (15.3)	56.9 (13.0)	55.0 (15.6)	45.6 (16.8)	48.0 (17.0)
Time between surgeries (months), mean (SD)		17.8 (14.4)	20.9 (18.2)	10.3 (4.9)	14.5 (10.2)	28.4 (17.7)
Tumor Type	Astrocytoma (Grade II-III)	6 (22.2)	3 (27.3)	3 (25.0)	0 (0.0)	0 (0.0)
	GBM (Grade IV)	20 (74.1)	8 (72.7)	8 (66.7)	2 (100.0)	2 (100.0)
	Gliosarcoma (Grade IV)	1 (3.7)	0 (0.0)	1 (8.3)	0 (0.0)	0 (0.0)
Initial treatment with TMZ, n (%)		26 (96.3)	11 (100.0)	2 (100.0)	11 (91.7)	2 (100.0)
TMZ Re-treatment at recurrence, n (%)		12 (44.4)	6 (54.5)	1 (50.0)	5 (41.7)	0 (0.0)
Initial treatment with RT, n (%)		26 (96.3)	11 (100.0)	2 (100.0)	11 (91.7)	2 (100.0)
Re-treatment with RT at recurrence, n (%)		8 (29.6)	2 (18.2)	1 (50.0)	5 (41.7)	0 (0.0)

## Discussion

In this study, we evaluated the longitudinal stability of MGMT promoter methylation in a large cohort of paired recurrent gliomas, integrating quantitative measurements with categorical status classification. We demonstrate that MGMT promoter methylation is quantitatively stable in most recurrent gliomas, with apparent status transitions occurring in a minority of cases with borderline methylation levels near classification thresholds. These results reinforce the clinical reliability of MGMT assessment at diagnosis while highlighting important limitations of binary reporting when quantitative methylation values approach cutoff boundaries.

The observed concordance rate of MGMT promoter methylation between recurrent tumors in this study aligns closely with prior reports, which have demonstrated stability in approximately 70–85% of cases [10–13]. Prior studies have shown intratumoral heterogeneity in MGMT methylation and have suggested that TMZ may exert selective pressure on distinct tumor subpopulations, potentially enriching compartments with distinct methylation distributions [21, 22]. Investigations have suggested that apparent gains or losses of MGMT hypermethylation may reflect this intratumoral heterogeneity, sampling variability, or selective pressure from alkylating chemotherapy rather than widespread epigenetic reprogramming [15–18]. Although our study cannot distinguish the contributions of these various testing and biological factors, incorporating quantitative methylation percentages provides context for interpreting apparent categorical transitions. Our analysis shows that tumors exhibiting categorical status changes generally harbored lower methylation levels at baseline or recurrence, consistent with epigenetic variability at the margins of classification rather than biologically distinct states.

These findings have direct implications for clinical practice, where MGMT promoter methylation is frequently reported as a dichotomous variable. While binary classification remains practical and widely adopted, it may obscure clinically relevant nuances in tumors with borderline methylation levels, which appear to represent a biologically less stable subgroup. Prior studies have similarly demonstrated that intermediate or low-level MGMT methylation is associated with less predictable response to TMZ and diminished prognostic value compared with clearly unmethylated or robustly hypermethylated tumors [15–18]. Our results further support the concept that MGMT methylation exists along a continuum and that quantitative reporting provides additional context, particularly when reassessing tumors at recurrence.

We also explored the relationship between MGMT methylation dynamics and the interval between paired sample collection, used here as a surrogate for disease course. Tumors that remained unmethylated demonstrated shorter intervals between sample collection compared with tumors that retained, gained, or lost hypermethylation. This finding is consistent with the established association between MGMT hypermethylation and improved response to alkylating chemotherapy and should be interpreted as reflective of underlying tumor biology rather than a causal effect of methylation transition itself [10–13]. Importantly, these analyses were descriptive and not intended to establish independent prognostic value for methylation changes over time. An exploratory analysis of tumor location revealed a higher proportion of frontal lobe tumors that remained hypermethylated at recurrence. While this observation is hypothesis-generating, it is consistent with prior work suggesting regional differences in MGMT methylation patterns and broader epigenetic landscapes within gliomas [23].

In a complementary, clinically annotated cohort, we examined how MGMT methylation status at recurrence related to real-world treatment decision-making. Nearly all patients received temozolomide following initial surgery, consistent with current standard-of-care practices [24]. At recurrence, temozolomide rechallenge was more frequently pursued in patients whose tumors remained hypermethylated, whereas alternative strategies, including re-irradiation or investigational therapies, were more commonly pursued in patients with consistently unmethylated tumors. Prior clinical trials and retrospective analyses have demonstrated greater benefit from TMZ rechallenge in patients with MGMT-hypermethylated tumors [19, 20]. Although our findings in this small clinical cohort are descriptive, they are aligned with the results of these previous studies and may reflect clinicians’ consideration of MGMT status at recurrence when selecting therapy.

Several limitations should be acknowledged. First, the retrospective nature of both cohorts introduces potential selection and sampling biases. Second, time between sample collection was used as a surrogate for disease course rather than formal progression-free survival, limiting causal interpretation. Third, treatment details in the commercial molecular database were unavailable, precluding assessment of treatment-specific effects on MGMT methylation dynamics or adjustment for therapy-related confounders. Finally, the clinical cohort was small and underpowered for survival analyses, and its findings should be considered exploratory. Despite these limitations, the large paired-sample size of the commercial cohort and the integration of quantitative methylation data represent key strengths of this study.

## Conclusion

MGMT promoter methylation remains quantitatively stable in most recurrent gliomas. Categorical status changes occur in a minority of cases largely confined to tumors with borderline methylation levels. These findings indicate that most apparent transitions in MGMT status reflect variability near classification thresholds rather than true epigenetic reprogramming. While MGMT assessment at diagnosis remains clinically reliable, reassessment at recurrence may provide additional value, particularly for tumors with borderline methylation. Quantitative methylation reporting may improve interpretation of MGMT status and better inform therapeutic decision-making at recurrence, including consideration of temozolomide rechallenge.

## Data Availability

The molecular profiling data analyzed in this study was provided by Caris Life Sciences through research collaboration and is not contained in a publicly accessible database. The authors may share the dataset used in this study upon approval from Caris Life Sciences. The data for the Rutgers Cancer Institute/RWJBarnabas Health and Lenox Hill Hospital clinical cohort is available from the corresponding author on reasonable request.
